# Supplementary material for: Effects of prolonged aerobic exercise and training intensity on memory cognition
Source: PLoS One. 2025 Feb 26;20(2):e0294381. doi: 10.1371/journal.pone.0294381 (PMC11864540; doi:10.1371/journal.pone.0294381)
Supplement: Attachment — Submitted filename: rebuttal letter.docx [file pone.0294381.s004.docx]

Dear Editor,

Thank you for the opportunity to revise our manuscript entitled "The Influence of Long-term Aerobic Exercise and Exercise Intensity on Exercise Memory Effect" submitted to PLOS ONE. We appreciate the insightful comments and suggestions provided by the reviewers, which have helped us to improve the quality of our work. Below is our point-by-point response to the reviewers' comments and the changes made in the revised manuscript.

**Response to reviewers**

**Reviewer 1:**

**Question1**:

The methodology description for assessing declarative and procedural memory is unclear (page 4). It seems unlikely that the procedure could be replicated solely from the provided text, restricting a fundamental aspect of science&mdash;study replication.

**Answer1:**

"The materials used in our experiment are primarily derived from declarative

knowledge related to the human blood circulation. These materials consist of 71 sentences

designed to comprehensively cover the subject matter, emphasizing the system's composition,

structure, function, and interrelationships among components. However, recognizing the

complexity of these relationships, we intentionally omitted explicit explanations for certain

relationships. To provide participants with more opportunities for reflection, we will further clarify

this point in the paper and provide additional details."

**Question 2:**

Although the study mentions evaluating declarative and procedural memory pre and post physical exercise intervention, it&#39;s unclear if different texts were used for student assessment at each stage.

**Answer2**:

The reviewer raised a question about whether different texts were used for assessing students' declarative and procedural memory and found this aspect unclear.

"Indeed, we used different texts to assess students' declarative and procedural memory before and after the exercise intervention. In Experiment 1, we employed declarative knowledge related to human blood circulation, while in Experiment 2, we shifted to procedural knowledge in geography education, specifically addressing the formation of air pressure and wind zones. We will emphasize this aspect more clearly in the paper to ensure a transparent understanding of the assessment process."

"Refer to the revision section in detail, specifically section 2.6 of the revised manuscript."

**Question 3**:

Additionally, the evaluation method for procedural memory and the distinction between &quot;Experiment 1&quot; and &quot;Experiment 2&quot; lack clarity. Is Experiment 1 the pre-test and Experiment 2 the post-test for declarative memory?

**Answer3:**

The reviewer expressed concerns about the distinction between Experiment 1 and Experiment 2 and whether they represent pre-test and post-test for declarative memory.

I see, Experiment 1 involves the assessment of declarative knowledge, while Experiment 2 focuses on procedural knowledge. Both knowledge assessments require pre- and post-tests, a clarification that I will elaborate on further in the manuscript。

"Refer to the revision section in detail, specifically section 2.6 of the revised manuscript."

**Question 4**:

How is procedural memory assessed? Is there a motor task involved? What does it entail?

**Answer4:**

About the assessment of procedural memory:For the evaluation, we employed specific tasks in the field of geographic education, mainly covering the following aspects:

Task types:"Experiment 2 aims to examine high school students' understanding of atmospheric pressure and the formation of wind zones. The assessment tasks include symbol comprehension, fill-in-the-blank exercises, picture interpretation, reasoning tasks based on visual stimuli, and discussions."

Procedural learning process:

"In the context of the origin of geography, the procedural learning process can be divided into three stages: acquisition, consolidation and transformation, and transfer and application. Our experiment focuses on three key aspects:"

Understanding of atmospheric structure:

"This aspect involves understanding the composition of the atmosphere, vertical stratification, atmospheric heating processes, thermal circulation principles, distribution of air pressure belts, wind zones, etc. Students are required to explain relevant phenomena, analyze the impact of air pressure belt and wind zone distribution on climate formation, and comprehend the causes of common weather phenomena through the principle of heat circulation."

Interpreting charts and images:

"Students provided schematic diagrams, including vertical wind layers in the atmosphere, insulation effects, thermal circulation patterns, global pressure zone distribution, wind zone formation and distribution, pressure zone charts, seasonal movement diagrams of wind zones, etc. We assessed their ability to interpret and analyze these diagrams, as well as their skills in drawing analysis and spatial positioning. Additionally, we examined students' logical reasoning abilities through explanations of phenomena based on the principle of thermal circulation."

Understanding of environmental and natural laws:

"This aspect emphasizes the significance of following the laws of nature and living in harmony with the environment, building upon an understanding of climate formation principles."

No, our procedural memory assessment does not involve specific motor tasks. Our research primarily focuses on the field of geographic education, evaluating students' understanding of concepts such as atmospheric pressure and the formation of wind zones. The assessment tasks mainly include symbol comprehension, fill-in-the-blank exercises, picture interpretation, reasoning tasks based on visual stimuli, and discussions. Students' tasks involve answering questions based on theoretical knowledge and charts, without the need for specific physical sports or motor tasks. We will provide further detailed explanations of our assessment approach in the paper to ensure readers have an accurate understanding of the experimental design.

It is important to note that we will provide further detailed explanations of these aspects in the paper to ensure readers have a clearer understanding of our procedural memory assessment approach.

"Refer to Section 2.5 for the introductory description of procedural knowledge measurement under Figure 1."

**Question 5:**

Beyond explanatory errors, there are challenging issues. Table 1 (page 3) mentions &quot;Cardiorespiratory Fitness Assessments,&quot; yet there are no indications of fitness evaluated.

**Answer5:**

At the beginning of each academic semester, students undergo a comprehensive health examination, including screening for cardiorespiratory function, as documented in the medical examination report. To ensure the suitability of students participating in this research for relevant physical activities and courses before the initiation of exercise intervention, they are required to provide information related to cardiorespiratory function from their medical examination reports. The purpose of this step is to verify the normalcy of students' cardiorespiratory function, ensuring their safe participation in the experiment. The information in these medical examination reports is sourced from specialized medical institutions to guarantee the objectivity and scientific rigor of the assessment.

“Refer to Section 2.2”.

**Question6:**

The absence of fitness evaluation leads to the use of generic intensity values that might not correspond accurately to the actual fitness levels, especially with a significantly large student cohort where fitness variability is expected to be high. Individual intensity thresholds, as defined in exercise physiology, can vary considerably.

**Answer6:**

While we mentioned "Cardiorespiratory Fitness Assessments" in Table 1, the nature of our study didn't involve providing detailed reports on the specific health conditions of each participant in the manuscript. Instead, we used cardiorespiratory fitness assessments as a screening tool to ensure that participants met the criteria for the experiment's intensity requirements. The purpose of this assessment was to identify students suitable for participation and ensure a reasonable range of health conditions. We will further clarify this point in the paper to elucidate our methodology and the key steps in the assessment.

Additionally, we acknowledge that this approach might lead to some generalization of intensity levels. In our discussion, we have already highlighted this potential limitation, emphasizing the variability that can exist in individual intensity thresholds according to exercise physiology. We will ensure to express these nuances more clearly in the final paper to address the reviewer's concerns.

**Question 7:**

Moreover, if the study presupposes that 8 weeks of cardiorespiratory exercise will enhance memory, shouldn&#39;t the study analyze whether there&#39;s been any cardiorespiratory improvement?

**Answer7:**

About your concern regarding the assessment of cardiorespiratory fitness, we understand your apprehension. Our study focuses on exploring the potential impact of exercise on memory rather than specifically assessing changes in cardiorespiratory health. The reason we did not conduct cardiorespiratory fitness assessments is that our primary emphasis is on changes in memory performance, and the specific effects on cardiorespiratory health fall beyond the scope of our study.

We want to emphasize that the purpose of our research is to investigate the relationship between exercise and memory, not to directly intervene in cardiorespiratory health. Future studies may consider including more detailed assessments of cardiorespiratory health to comprehensively understand the potential benefits of exercise on the body. We will carefully consider your suggestions and explicitly address the limitations of our study in the discussion to ensure that readers have a clearer understanding of our research design and objectives.

**Question 8:**

While the study identifies improvements in declarative memory, are they solely due to physical exercise practice or a change in fitness? If improvements are only related to activity, why wait for 8 weeks?

**Answer8:**

We appreciate your concerns, and from a scientific perspective, there is substantial evidence supporting a positive correlation between the duration of exercise intervention and improvements in cognitive function. Long-term exercise interventions are believed to facilitate lasting changes in neural plasticity and sustained improvement in brain function.

Previous research suggests that improvements in cognitive function, including memory, may take some time to manifest, and longer-term exercise interventions provide more extensive opportunities for these effects to become apparent. For instance, studies indicate that structural and functional changes in the brain may require a relatively extended period, further supporting the effectiveness of long-term exercise interventions.

Furthermore, research indicates that the impact of exercise on cognitive function may involve complex interactions across multiple physiological and neural systems, and these effects may take time to manifest at the cognitive level. Therefore, choosing an 8-week period for the exercise intervention is aimed at allowing sufficient time to observe the potential emergence of these effects.

We believe that through a more extended period of exercise intervention, we are likely to observe more enduring and significant improvements in memory, which can have positive implications for students' long-term cognitive health and academic achievements. We will extensively discuss the scientific support for this in the paper to better explain the rationale behind our study design and the expected outcomes.

**Question9:**

Regarding procedural memory, the lack of details in the method hinders thorough evaluation.

**Answer 9:**

Regarding details on procedural memory:We appreciate the reviewer's attention to the methods section. We will provide more detailed information on procedural memory in the revised manuscript to ensure transparency and reproducibility of the methods.

**Response to minor issues:**

Question：Figures 3, 4, and 5 will be revised to include standard deviations.

Answer:

In the field of psychological statistics, it is customary to present comprehensive numerical data, including standard deviations, in data tables. This presentation method is advantageous for readers to conduct in-depth analysis and understanding of the specific data conditions. In contrast, when it comes to figures, our emphasis lies in the visual representation of data to ensure clarity and simplicity, facilitating readers in intuitively grasping data trends and key information.

Therefore, we avoid excessive presentation of detailed numerical data in figures to maintain readability and conciseness. It's worth noting that the data tables in our paper provide a clear presentation of standard deviations. We appreciate your valuable suggestions and thank you for bringing this to our attention.

Question:Post-hoc analyses will be conducted for a clearer presentation of changes.

Answer:

Thank you for your attention to our study and your valuable suggestion regarding post-hoc tests. In response to your query, we would like to clarify that in analyzing the interaction effect, we opted for simple effects analysis and simple simple effects analysis to comprehensively explore the impact between different levels of factors.

Simple effects analysis allows us to examine whether one factor has a significant effect on the dependent variable at a specific level of another factor. The extension to simple simple effects analysis further enables us to test the significance of effects across all possible combinations of factor levels.

We believe that the choice of these two analysis methods provides a more thorough and comprehensive understanding, particularly when dealing with situations involving interaction effects.

Question：On page 1, The abstract requires rewriting due to inherent repetitiveness.

Answer：The abstract will be rewritten to address repetitiveness.

Question:On page 1, Citations supporting claims are missing from lines 7 to 17 in the introduction.

Answer：Citations will be added in the introduction to support claims (lines 7-17).

Question:On page 2, In the introduction, Shephard is cited without the year, assuming it is the second reference in the bibliography, but this needs to be clarified.

Answer: Shephard's citation will be clarified, including the publication year.

Question:The bibliography lacks ordering and needs to be alphabetized for author traceability.

The bibliographic format needs to be clarified.

Answer:The bibliography will be alphabetized for author traceability and the format clarified.

Question；

On page 4, In the section &quot;2.5 Measure tools,&quot; Chi et al. lack a publication year.

In the Discussion, BEST and MCMORRIS are cited in uppercase.

Paragraph spacing in the discussion appears arbitrary and inconsistent at times.

Answer:The citation for Chi et al. in the "2.5 Measure tools" section will include the publication year.

Paragraph spacing in the discussion will be adjusted for consistency.

**Reviewer 3:**

**Question1**:

Abstract

The opening sentences is too strong, make it more consistent with your actual work. After all you are only studying healthy young people and this does not apply to other populations.

Please add numbers to support you claims, this now looks very vague. Also add a conclusion to this paragraph, by identifying the magnitude of the effect(s).

**Answer1:**

Opening Sentences:

Reviewer's Comment: "The opening sentences are too strong, make it more consistent with your actual work."

Your Response: "Thank you for the feedback. We appreciate your perspective. In light of your suggestion, we will revisit the opening sentences to ensure that they align more accurately with the nuances of our study, particularly focusing on the population under investigation, which comprises healthy young individuals."

Adding Numbers for Support:

Reviewer's Comment: "Please add numbers to support your claims; this now looks very vague."

Your Response: "We acknowledge the importance of providing concrete numerical support for our claims. In the revised manuscript, we will incorporate specific numerical results to substantiate the observed effects, thereby addressing the perceived vagueness."

Conclusion in the Abstract:

Reviewer's Comment: "Also, add a conclusion to this paragraph by identifying the magnitude of the effect(s)."

Your Response: "Your suggestion to include a concluding statement in the abstract is valuable. We will ensure that the revised abstract includes a succinct conclusion that highlights the magnitude of the observed effects, offering readers a clearer understanding of the study's impact."

Remember to express appreciation for the reviewer's input and convey your commitment to addressing their concerns in the revised manuscript.

**Question2**:

Introduction

You need to reference the opening line. In general, the few opening sentences and the message they deliver - well known and even present in newspapers. Try please to write this from a more scientific angle.Add study hypothesis here.

**Answer2:**

Introduction:

The opening statements in the introduction may benefit from a more tempered tone, aligning with the study's focus on healthy young individuals rather than making broad claims applicable to diverse populations. Furthermore, inclusion of a study hypothesis would enhance the introduction's clarity.

Revised Introduction:

Cognitive psychologists categorize knowledge into declarative and procedural types. Declarative knowledge concerns "what" something is, while procedural knowledge relates to "how" to perform a task (Anderson, 1976). The latter builds upon the former, supplying information for task execution. In the context of learning, students primarily acquire procedural knowledge in its declarative form (Xia, 2000). This foundational role of knowledge in motor learning contributes significantly to knowledge mastery and, consequently, overall student development.

The connection between knowledge acquisition and students' holistic well-being, both physically and mentally, has drawn attention across educational, psychological, and sports science domains. Previous studies in sports science have indicated a positive impact of physical exercise on academic performance. However, the extent to which different exercise intensities affect learning outcomes remains unclear, necessitating a comprehensive examination.

Exercise intensity, defined as the degree of physiological stimulation from physical activities, plays a crucial role in students' physical and mental development (Chen et al., 2014). Current research has primarily focused on the impact of exercise on learning outcomes, lacking comprehensive empirical studies on the effects of different exercise intensities. Therefore, this study hypothesizes that moderate and low-intensity exercise can influence learning outcomes by affecting the process of acquiring procedural and declarative knowledge.

**Question 3:**

Methods

There are some typo&rsquo;s and extra space(es) that you should amend here.

Add standard deviations in Table 1.

So highlight that this was a cross-sectional comparative study.

Please reference the ACSM guidelines.

VO2 should be presented as V̇O2, as this is the correct way to indicate flow, here and throughout the MS.

Could you please outline which Polar device you were using, and also add data on sampling rate and data extraction.

Please reference Chi et al.

Figures 1 and 2 should be given as Supplementary data, since this is not your original work.

More data on SPSS 23.0 are warranted here.

Mechanisms in this specific population of students, not in general population. And second, this is a very speculative conclusion since its quite difficult to make such strong conclusions based on the findings of a cross sectional study. Please implement this into your results section.

Figures 3 to 5.

The title of the Y-axis should be re-arranged (wrong direction).

**Answer3:**

Methods:

Spelling errors and extra spaces: We will thoroughly review the entire manuscript, correcting all spelling errors and extra spaces to ensure grammatical and spelling accuracy.

Add standard deviations in Table 1: We will add the corresponding standard deviations to Table 1 to provide more comprehensive data information.

Emphasize that this is a cross-sectional comparative study: In the methods section and where appropriate, we will explicitly highlight that this study is a cross-sectional comparative study to avoid any misinterpretation.

Reference ACSM guidelines: We will cite the guidelines from the American College of Sports Medicine (ACSM) at relevant points to support the content in our study.

Present oxygen consumption as V̇O2: Throughout the manuscript, we will use the correct symbol (V̇O2) to represent oxygen consumption.

Provide detailed information on the Polar device and add data details: In the methods section, we will offer more detailed information about the Polar device used, including sampling rate and data extraction, ensuring readers have a clear understanding of the experimental details.

Cite the literature by Chi et al.: We will include a citation to the work by Chi et al. to support the background of our study.

Present Figures 1 and 2 as Supplementary data: We will place Figures 1 and 2 in the Supplementary data section to clarify that they are not original works.

Offer more data on SPSS 23.0: In the methods section, we will provide additional information about the use of SPSS 23.0 to enhance transparency.

Speculative nature of mechanisms and conclusions in this specific student population: In the results section, we will more accurately describe potential mechanisms in this specific student population and temper speculative conclusions to align with the limitations of a cross-sectional study.

Reorganize the Y-axis titles in Figures 3 to 5: We will rearrange the Y-axis titles in Figures 3 to 5 to ensure accuracy.We chose the current order of the Y-axis and X-axis based on our intention to highlight the performance of different groups across various scores, emphasizing the focus of our research. We believe that this layout intuitively presents our findings and contributes to a better understanding of the research results by the readers.

Through these modifications, we aim to enhance the scientific rigor and clarity of the manuscript in response to the reviewer's suggestions and expectations.

We appreciate the valuable suggestions from the reviewer and will actively consider these recommendations in the revision process to enhance the quality and comprehensibility of the paper. If you have any further questions or need more details, please feel free to ask.

**Question 4:**

Discussion

Then please reference these studies.

Why is reference BEST written in capital letters ? MCMORRIS T, 2009 as well ??

In general, your discussion section is often too long and sometimes too vague. You should focus

on your results and interpret them in the context of what was done before. Also, you have too

many smaller paragraphs and its difficult for a reader to follow the context. These should be

structured in a more comprehensive manner.

**Answer 4:**

Citation Format:

Reviewer's Question: Why are BEST and MCMORRIS cited in uppercase?

Response: We will rectify the citation format for BEST and MCMORRIS, using the correct citation style and ensuring consistency throughout the manuscript.

Discussion Structure Issue:

Reviewer's Concern: Your discussion section is often too long and sometimes too vague. You

should focus on your results and interpret them in the context of what was done before. Also, you

have too many smaller paragraphs, and it's difficult for a reader to follow the context. These

should be structured in a more comprehensive manner.

Response: We will reorganize the discussion section to present research results more succinctly

and clearly within the context of previous studies. We will consolidate relevant smaller paragraphs

to improve overall structure, making it easier for readers to understand and follow our argument.

Citation of Studies:

Reviewer's Suggestion: Reference these studies in the discussion.

Response: We will appropriately cite relevant studies in the discussion section to better support

our conclusions and ensure a clearer connection with the existing literature.

Through these modifications, our aim is to enhance the scientific rigor, clarity, and overall

coherence of the article, addressing the reviewer's suggestions and improving the overall quality.

Combining the feedback from both reviewers, the main focus has been on the clarity of the

methodology, correction of spelling errors, enhancement of table data, and the structure and

conciseness of the discussion section. They have provided constructive suggestions, and

addressing these areas will contribute to improving the readability and scientific quality of the

paper. We appreciate the guidance from the reviewers and will diligently implement modifications

according to each suggestion to ensure comprehensive improvement in these aspects of the

manuscript.

Yours Sincerely,

Dr. Li Xinan

Zhong Huihe
