# Supplementary material for: Effects of prolonged aerobic exercise and training intensity on memory cognition
Source: PLoS One. 2025 Feb 26;20(2):e0294381. doi: 10.1371/journal.pone.0294381 (PMC11864540; doi:10.1371/journal.pone.0294381)
Supplement: Attachment — Submitted filename: Response to Reviewers9.26.doc [file pone.0294381.s005.doc]

Dear Editor and Reviewers,

First and foremost, we would like to express our sincere gratitude for the time and effort you have dedicated to reviewing our manuscript and for providing valuable comments and suggestions. We greatly appreciate the meticulous work and professional feedback you have offered. Your insights have been immensely helpful in enhancing the quality of our research and in refining the content of our paper.

We have carefully considered each of the reviewer comments and have responded to them individually in the following text. We have made corresponding revisions to the manuscript to address the concerns raised by the reviewers and to improve the clarity and academic rigor of the paper.

We believe that, with these modifications, our study can more accurately convey our findings and provide valuable insights to the academic community. Below are the specific responses to the reviewer comments and the revisions we have made:

Reviewer #3: I would like to thank the authors for following my comments and suggestions. Just one recommendation, please give a final version of this MS to a native speaker.

Response:

Thank you for your review of our manuscript and the valuable comments and suggestions provided. We have carefully considered your recommendations and plan to have the final version of the manuscript proofread by a native English speaker to ensure the quality and accuracy of the language and expression.

Reviewer #4:

1.The term "exercise" is repeated multiple times in the research title, and it is suggested to simplify it to avoid redundancy and to enhance the clarity and impact of the title.

Response:

The title is changed to"Effects of Prolonged Aerobic Exercise and Training Intensity on Memory Cognition"

2. In the introduction and discussion sections, it is essential to thoroughly explain the relationship between physical activity and cognitive functions.

Response:

I have incorporated the suggestions from the reviewers into the revised manuscript, specifically in the Introduction and Discussion sections. The changes have been made in response to the feedback provided and are detailed in the attached revised manuscript.

Reviewer #5:

1.Clarity of Methodology

The methodology, particularly regarding the assessment of declarative and procedural memory, lacks clarity. It is essential for the methodology to be described in a way that allows for replication by other researchers.

Response: We appreciate the reviewer's feedback. We recognize the importance of a clear methodology for scientific research. In the revised manuscript, we have significantly expanded the methods section to include detailed steps, tools used, and specific procedures. Furthermore, we have provided the source of all test materials to ensure that other researchers can replicate our experiments.For specific details, please refer to the "Methods" section of the revised manuscript.

2.The manuscript mentions "cardiopulmonary health assessments" but does not provide detailed evaluations of fitness levels.

Response: In our revised manuscript, we have included a comprehensive evaluation of cardiopulmonary fitness. Specifically, we have detailed the criteria used to determine the fitness levels of the participants based on their physical examination reports. Only students whose reports from the university health center indicated normal cardiopulmonary function and who met the established health standards were included in the study. This additional information ensures that the readers have a clear understanding of the health assessment process and the fitness levels of the participants included in our research.

3.Unclear Research Hypothesis

The introduction does not clearly state the study hypothesis.

Response: Thank you for the reminder. We have now clearly stated the research hypotheses in the introduction and structured the entire research framework around these hypotheses. We have also provided a detailed explanation of the theoretical basis and research design for these hypotheses.

4.Data variability is lacking: Figures 3, 4, and 5 do not include standard deviations, which are necessary for understanding the variability of the data. Including this data will enhance the rigor of the statistical analysis.

Response:

The standard deviation in the figure is clearly labeled as "Standard Deviation."

5. There are typographical errors and inconsistencies in formatting.

Response: We have conducted a thorough review of the entire manuscript, correcting all typographical errors and formatting inconsistencies. We have also employed professional typesetting software to ensure the document's consistency and professionalism.

6..The discussion section is verbose and vague.The discussion section is overly lengthy and often unclear.

Response: We have streamlined the discussion section to make it more focused and concise. We have ensured that the discussion closely revolves around the results and is interpreted in the context of existing literature. Additionally, we have reduced speculative language to ensure that all conclusions are supported by data.
