# Supplementary material for: Effects of prolonged aerobic exercise and training intensity on memory cognition
Source: PLoS One. 2025 Feb 26;20(2):e0294381. doi: 10.1371/journal.pone.0294381 (PMC11864540; doi:10.1371/journal.pone.0294381)
Supplement: Attachment — Submitted filename: Plos one revision.docx [file pone.0294381.s002.docx]

Thank you for the opportunity to revise the current MS. Please find my comments, line by line.

The first thing that caught my attention was the fact that line numbers were missing. Revising a manuscript with no line numbers was quite challenging and often results in communication issues among authors and reviewers. Please add line numbers to this MS.

Abstract

The opening sentences is too strong, make it more consistent with your actual work. After all you are only studying healthy young people and this does not apply to other populations.

Please add numbers to support you claims, this now looks very vague. Also add a conclusion to this paragraph, by identifying the magnitude of the effect(s).

Introduction

You need to reference the opening line. In general, the few opening sentences and the message they deliver - well known and even present in newspapers. Try please to write this from a more scientific angle.

Add study hypothesis here.

Methods

There are some typo’s and extra space(es) that you should amend here.

Add standard deviations in Table 1.

So highlight that this was a cross-sectional comparative study.

Please reference the ACSM guidelines.

VO2 should be presented as V̇O_2_, as this is the correct way to indicate flow, here and throughout the MS.

Could you please outline which Polar device you were using, and also add data on sampling rate and data extraction.

Please reference Chi et al.

Figures 1 and 2 should be given as Supplementary data, since this is not your original work.

More data on SPSS 23.0 are warranted here.

Mechanisms in this specific population of students, not in general population. And second, this is a very speculative conclusion since its quite difficult to make such strong conclusions based on the findings of a cross sectional study. Please implement this into your results section.

Figures 3 to 5.

The title of the Y-axis should be re-arranged (wrong direction).

Discussion

Then please reference these studies.

Why is reference BEST written in capital letters ? MCMORRIS T, 2009 as well ??

In general, your discussion section is often too long and sometimes too vague. You should focus on your results and interpret them in the context of what was done before. Also, you have too many smaller paragraphs and its difficult for a reader to follow the context. These should be structured in a more comprehensive manner.
