# Supplementary material for: Effects of prolonged aerobic exercise and training intensity on memory cognition
Source: PLoS One. 2025 Feb 26;20(2):e0294381. doi: 10.1371/journal.pone.0294381 (PMC11864540; doi:10.1371/journal.pone.0294381)
Supplement: Attachment — Submitted filename: Response to Reviewers10.18 .docx [file pone.0294381.s006.docx]

Response to Reviewers

Dear Reviewer,

Thank you very much for your thorough review and constructive feedback on our manuscript. We have taken your comments seriously and have made the following revisions to address the concerns raised:

Q1.Clarification of Theoretical Framework and Hypotheses:

Answer1:We have expanded the introduction to provide a more detailed explanation of the theoretical framework underpinning our study. We have also bolstered our rationale for examining the effects of different exercise intensities on declarative versus procedural knowledge with additional literature support.

Q2.Enhanced Methodology Details:

Answer2:The methodology section has been revised to include more comprehensive details, particularly regarding the assessment tools for declarative and procedural knowledge. We have described the validity, reliability, and specific adaptations made to these measures for our study.The recruitment and selection process for participants has been detailed in the "Methods" and "Grouping" sections of our manuscript, ensuring the transparency and scientific rigor of the participant selection.

Q3.Improved Statistical Analysis Explanation:

Answer3:Effect sizes are indicated in the corresponding statistical analysis results, for example, below Table 3.

Q4.Streamlined Discussion Section:

Answer4:We have condensed the discussion to focus more precisely on the findings in relation to the original research questions and the existing body of literature.

Q5.Formatting and Typographical Consistency:

Answer5:The manuscript has undergone careful proofreading, and all formatting and typographical inconsistencies have been corrected to ensure the professional presentation of our work.

Q6.Focused Conclusion Section:

Answer7:The conclusion has been revised to explicitly state the key findings and their implications for sports science and educational practice.
