# Supplementary material for: Effects of prolonged aerobic exercise and training intensity on memory cognition
Source: PLoS One. 2025 Feb 26;20(2):e0294381. doi: 10.1371/journal.pone.0294381 (PMC11864540; doi:10.1371/journal.pone.0294381)
Supplement: Attachment — Submitted filename: Response to Reviewers.docx [file pone.0294381.s007.docx]

**Response to Reviewers**

**Response to the Academic Editor's Inquiry:**

Q1：Firstly, the gender imbalance in your sample (191 males vs. 378 females) is particularly worrying. This disparity may skew your findings and undermine the results unless adequately addressed through appropriate statistical controls.

Answer1:

In this study, we noted an uneven gender distribution in the sample, which is consistent with the overall gender ratio of our college, where there are more females than males. Although our sample reflected this gender imbalance, when we conducted a 3 (exercise intensity) × 2 (knowledge test time) × 2 (gender) analysis of variance (ANOVA), we found that the interaction between gender, exercise intensity, and knowledge measurement time was not significant. This suggests that, within the current study's design and analytical framework, gender differences did not significantly affect learning outcomes.

Nevertheless, we recognize that the gender distribution in the sample may have affected the generalizability of the study results. To further explore the potential impact of gender differences, we suggest that future research should strive for a more balanced gender representation or employ more sophisticated statistical methods to control for potential biases in gender distribution. Additionally, we consider this characteristic of gender distribution as a limitation of the present study and have thoroughly considered the possible impact of this factor in the discussion section.

In the limitations section of this study, we further discuss the potential impact of the uneven gender distribution on the interpretability of the study results and emphasize the need for deeper exploration of this issue in future research. We believe that although gender differences did not significantly affect the main results in this study, this finding does not entirely rule out the role that gender factors may play in other research contexts or with different samples. Therefore, we encourage subsequent researchers to continue to pay attention to the potential impact of gender differences on research results and to adopt appropriate methodological strategies to address this issue.

Q2:A Rational Explanation for the Verification of Exercise Intensity:

Answer2:

In this study, The RS800CXSD heart rate telemetry monitor (manufactured in Finland was used to monitor varying intensities of aerobic exercise loads.

Through this dual monitoring approach, we were able to monitor and record participants' heart rate changes in real-time, ensuring precise control of the exercise intensity.

We understand your concern regarding the rigor of the verification process. To enhance the transparency and reproducibility of our research, we have included additional details in the revised manuscript. Specifically, we have detailed the model of the Polar heart rate monitor used and provided an exhaustive description of the monitoring frequency and data recording procedures. Moreover, we have supplied an intensity monitoring record form for participants, which illustrates the real-time fluctuations in heart rate data throughout the study, thereby enhancing the credibility of our data.

**Response to the Reviewer #5:**

Q1:Gender Distribution Imbalance:

Answer1:Thank you for highlighting the issue of gender distribution. In the revised manuscript, we have included an in-depth analysis of the potential impact of gender distribution on the results and have thoroughly considered this factor's possible influence in the discussion section. Although the gender interaction was not significant, we still explored the potential impact of gender differences using advanced statistical methods.

Q2：Insufficient Theoretical Basis:

Answer2:Your question about the theoretical basis for the intervention duration is crucial. We have included more relevant studies in the revised manuscript to support the theoretical basis for our chosen 8-week intervention period, ensuring that our study design is scientifically sound.

Q3:Inadequate Control of Confounding Variables:

Answer2:We recognize the insufficiency in controlling potential confounding variables. In the revised manuscript, we have added control for sleep, nutrition, and academic workload and considered these variables in our analysis to reduce their potential impact on the results.

Q4:Lack of Detail in Heart Rate Monitoring Method:

Answer4：Thank you for your attention to the heart rate monitoring method. In the revised manuscript, we have detailed the device model (Polar H10) used for heart rate monitoring and included a graph of the intensity changes of the participants, as well as how we ensured the accuracy of heart rate data through a combination of Polar heart rate telemetry and manual radial artery pulse measurements.

Q5:Statistical Analysis Issues:

Answer5:We appreciate your focus on statistical analysis. In the revised manuscript, we have ensured adequate testing of MANOVA assumptions, consistently reported effect sizes, and included statistical power analysis. We have also explored interaction effects in-depth and considered gender as a covariate.

Q6:Figures and Reporting Issues:

Answer6:We have updated all figures to include error bars and statistical notations and have ensured consistent reporting of effect sizes throughout the manuscript to improve data transparency and interpretability.

Q7:Discussion and Methodological Clarification:

Answer7:We have provided a more detailed discussion of gender-specific findings, mechanistic explanations, and practical implications, and have clarified the methodological section, including participant exclusion criteria and standardization of skill assessments, to enhance the transparency and reproducibility of the study.

Q8:Technical Issues:

Answer8:Thank you for your suggestions. In the revised manuscript, we have included reliability coefficients for the knowledge assessment tools and improved the documentation of activities. Additionally, we have reported the environmental conditions during testing to ensure the rigor of the study.

Q9:Suggestions for Improvement:

Answer9:We have taken your advice and have added physiological validation, strengthened statistical methods, controlled confounding variables, and provided a detailed reliability analysis of assessment tools in the revised manuscript to improve the quality and credibility of the study.
